# IL-6 Receptor Antagonists and Severe Post-COVID-19 Outcomes: An Emulated Target Trial

**DOI:** 10.64898/2026.02.27.26347274

**Published:** 2026-03-02

**Authors:** Zachary Butzin-Dozier, Manav Kumar, Yunwen Ji, Lin-Chiun Wang, A. Jerrod Anzalone, Eric Hurwitz, Rena C. Patel, Rachel Wong, Carolyn Bramante, Benjamin Sines

**Affiliations:** 1 School of Public Health, University of California, Berkeley, Berkeley, CA USA; 2 University of Nebraska Medical Center, Omaha, NE, USA; 3 University of North Carolina at Chapel Hill, Chapel Hill, NC, USA; 4 University of Alabama at Birmingham, Birmingham, AL, USA; 5 Renaissance School of Medicine, Stony Brook University, New York, NY, USA; 6 University of Minnesota, Minneapolis, MN, USA

## Abstract

**Background:**

Interleukin-6 (IL-6) is a cytokine that plays a key role in systemic hyperinflammation and may mediate the relationship between acute COVID-19 and severe long-term outcomes such as Long COVID or death. IL-6 modulating drugs may reduce patients’ risk of severe post-COVID-19 outcomes.

**Methods:**

We conducted an emulated target trial in a retrospective cohort of patients with moderate-to-severe rheumatoid arthritis who were prescribed IL-6 receptor antagonists (sarilumab or tocilizumab, pooled treatment) or other biologic agents (anakinra or baricitinib, pooled comparator) in 2022. We compared the 12–month cumulative incidence of mortality and Long COVID (diagnosed and probable) between groups using Super Learner and targeted maximum likelihood estimation, adjusting for covariates of interest.

**Results:**

In our cohort of 3,553 patients, we found that prescription of IL-6 receptor antagonists was associated with a lower 12-month cumulative mortality (adjusted relative risk (aRR) 0.40, 95% CI 0.27, 0.59), diagnosed Long COVID aRR 0.42, 95% CI 0.23, 0.78), and probable Long COVID (aRR 0.71, 95% CI 0.61, 0.83), compared to prescription of other biologic agents, among rheumatoid arthritis patients.

**Conclusions:**

IL-6 receptor antagonists may prevent the incidence of severe post-COVID-19 outcomes, such as Long COVID or mortality. This supports the hypothesis that IL-6 may be a mechanistic biomarker of COVID-19 sequelae and that acute COVID-19 severity may mediate this relationship.

Patients with prolonged symptoms of COVID-19 have persistent inflammatory dysregulation, and both Long COVID and acute COVID-19 are associated with dysregulation of cytokines, particularly interleukin (IL)-6. ^1–5^ Community cohort studies have demonstrated elevated IL-6 levels in patients with Long COVID compared with those who have fully resolved symptoms after acute SARS-CoV-2 infection.^5,6^ Furthermore, elevated IL-6 levels during inpatient treatment of acute COVID-19 are associated with a twofold increase in the risk of Long COVID compared with normal IL-6 levels at the time of acute infection.^4^

Investigators have proposed mechanistic causal models that support IL-6 as a mediator of the relationship between acute infection and Long COVID.^1–5,7–10^ IL-6 may mediate long-term neurological symptoms of COVID-19 infection by disrupting T helper 17 and regulatory T cell responses, thereby contributing to sustained inflammatory dysfunction and ultimately leading to fatigue.^10^ This inflammatory milieu has been hypothesized to induce an inflammatory feedback loop mediated by a persistent and exaggerated cellular immune response to cytokine release or chronically elevated cytokine release.^1^

These findings have motivated the hypothesis that IL-6 modulating medications, such as tocilizumab and sarilumab, may reduce the risk of Long COVID. Both randomized and observational studies have generally supported that IL-6 receptor antagonists, sarilumab and tocilizumab, may reduce mortality among COVID-19 patients, although further investigation is needed regarding their impact on Long COVID.^11–15^

This study seeks to evaluate the relationship between prescription to IL-6 receptor antagonists (sarilumab or tocilizumab) vs. prescription to other biologic agents (anakinra or baricitinib) and the subsequent risk of severe post-COVID-19 outcomes in a sample of patients with rheumatoid arthritis.

## METHODS

### Sample:

This study included electronic health record data for patients in the National Clinical Cohort Collaborative (N3C). N3C offers a rich, high-dimensional data source for a national sample of patients in order to make meaningful inferences regarding the long-term sequelae of COVID-19. N3C includes more than 31 billion rows of data and 8 million COVID-positive patients and 14 million demographically matched controls from 83 data-providing health institutions.^16,17^ N3C identifies patients with acute COVID-19 infection as patients who had either (1) at least one laboratory test with a positive result, (2) at least one “strong positive” diagnostic code in ICD-10 or SNOMED, (3) at least two “weak positive” diagnostic codes in ICD-10 or SNOMED, and selects two demographically-matched controls for each case.^18^ We sampled patients from N3C regardless of past or future COVID-19 status (i.e., including both cases and controls) to avoid inducing bias by restricting on a factor that is an effect of the exposure of interest.^19–24^

### Restriction:

We restricted to only include data sites that meet a minimum threshold of reporting for the exposure and outcome of interest (immune modulator prescription and Long COVID) among our target population, defined by more than one standard deviation below the mean reporting rate. These sites have a high risk of (potentially differential) misclassification, and this method is consistent with previous literature.^25,26^

### Temporal Window:

We included patients in N3C with a diagnosis of moderate-to-severe rheumatoid arthritis (severity inferred from prescription of a biologic agent) who were prescribed a study drug between December 31, 2021, and January 1, 2023. This ensures that (1) all person-time at risk for Long COVID took place after the release of the Long COVID diagnostic code (ICD code U09.9, “post COVID-19 condition, unspecified,” released October 1, 2021),^27^ (2) COVID-19 infections took place during the Omicron period (supporting consistency in viral and diagnostic trends),^25,28,29^ (3) all patients had equivalent (12 months) outcome monitoring time (i.e., no administrative censoring).

### Inclusion Criteria:

We included patients with moderate or severe rheumatoid arthritis (as mild rheumatoid arthritis would not indicate prescription of a biologic agent) who were prescribed one of the following biologic agents, tocilizumab, sarilumab, baricitinib, or anakinra during the study enrollment period and had not been prescribed another study drug in the previous three months. We excluded patients who were prescribed a study drug for severe COVID-19 infection.

### Covariates:

We included the following individual-level covariates that may be related to rheumatoid arthritis, provider prescribing decisions, COVID-19 severity, and Long COVID: healthcare utilization rate (described further below), sociodemographic information (sex, age at acute COVID-19 infection, race and ethnicity), comorbidities (bipolar disorder, immunocompromised status, body mass index, diabetes (complicated and/or uncomplicated), chronic lung disease, congestive heart failure, heart failure, acute kidney injury, myocardial infarction, hypertension, asthma, depression, Charlson Comorbidity Index), medication and tobacco usage (tobacco smoking status, systemic corticosteroids), COVID-19 related factors (total number of COVID-19 vaccination and booster doses, indicators of COVID-19 vaccination 0 to 6 months and 6 to 12 months before enrollment), and date of enrollment (prescription).^30,31^ In addition, we adjusted for the following county-level socioeconomic covariates, given the lack of individual-level socioeconomic information in N3C: percent of households with income below the federal poverty line and the social vulnerability index. As healthcare utilization is highly associated with Long COVID diagnosis, we accounted for heterogeneous healthcare utilization in multiple ways. We adjusted for baseline (prior to the exposure of interest, i.e. enrollment) healthcare utilization, defined as the monthly healthcare visitation rate (healthcare visits per month from December 2018 [beginning of N3C observation period] to enrollment). In addition, we considered healthcare utilization during follow-up as a source of informative censoring (i.e., truncation), and we evaluated the counterfactual impact of the exposure of interest, given that all participants were monitored (i.e., had a healthcare interaction) at least once during the 12 months of follow-up.^30,32–34^

### Exposures:

Exposures of interest include prescription to tocilizumab or sarilumab (monoclonal antibodies to IL-6), baricitinib (a small molecule inhibitor of JAK2), or anakinra (an IL-1 receptor antagonist), which are FDA-approved immune-modulating biologic agents.^35^ These immune modulators have all received FDA emergency use access or full approval for the treatment of severe COVID-19-related respiratory disease and have been used broadly throughout the pandemic.^36^ Additionally, these medications are FDA-approved for the treatment of rheumatoid arthritis with persistent symptoms despite disease-modifying anti-rheumatic therapy.^35^ As a result, there is a subset of patients with chronic exposure to these targeted immune-modulating therapies who were subsequently infected with SARS-CoV-2. We considered tocilizumab and sarilumab to be the “intervention” drugs, while baricitinib and anakinra were active comparators.

### Outcomes:

We evaluated 12-month cumulative incidence of (1) diagnosis of Long COVID based on U09.9 diagnosis code,^27^ (2) probable Long COVID, and (3) mortality as the primary outcomes of interest. The Long COVID computational phenotype is a validated measure that calculates probable Long COVID using individual EHR metrics, and previous studies have used a threshold of 0.9 to indicate probable Long COVID. ^26,37–41^ We evaluated COVID-19 incidence and severe COVID-19 incidence as secondary outcomes. We defined “severe” COVID-19 as any patient who was hospitalized, received invasive ventilation, or died due to COVID-19.

### Analysis Methods:

This analysis used an active comparator new user design.^42,43^ First, we applied Super Learner to maximize prediction of the outcome, given treatment and covariates (outcome regression), and model the probability of treatment (prescription of monoclonal antibodies to IL-6, compared to baricitinib and anakinra) given individual covariate status (i.e., treatment mechanism). We included the following nonparametric and nonparametric candidates in our Super Learner library to minimize the risk of model misspecification: generalized linear models (“SL.glm”), GLM net (“SL.glmnet”), and XGBoost (“SL.xgboost”).^44^ Super Learner is well-suited to this data setting, where EHR allows investigators to adjust for high-dimensional covariate data (i.e., many covariates per individual), which would lead to model misspecification via traditional parametric analysis approaches.^22,44,45^ We applied targeted maximum likelihood estimation to analyze the impact of the prescription of monoclonal antibodies to IL-6, compared to baricitinib and anakinra, on the 12-month cumulative incidence of Long COVID among individuals with rheumatoid arthritis.^20,22^ Targeted maximum likelihood estimation is a bias reduction tool that allows us to incorporate causal inference considerations of confounding and temporality to evaluate our causal parameter of interest. Our primary analysis evaluated treatment based on the first drug prescribed during the study period (emulating an intention-to-treat design). We considered death or lack of healthcare utilization (i.e., no healthcare interactions in 12 months after treatment initiation) as informative censoring, and we estimated the counterfactual impact of the intervention under a scenario of universal observation (i.e., at least one healthcare interaction during outcome period and no death [except for analyses of death as the primary outcome of interest]).^34^

### Secondary Analysis:

We evaluated the relationship between immune modulator use and severe post-COVID-19 outcomes, restricting to patients with documented COVID-19 prior to enrollment (i.e., prescribed study drug after acute COVID-19) and adjusting for COVID-19 severity (4-point ordinal score). This secondary analysis is analogous to the controlled direct effect of immune-modulating drugs on severe post-COVID outcomes among patients with COVID-19, excluding the indirect pathway mediated through Long COVID incidence and severity.^46^ In other words, this secondary analysis evaluated whether the impact of immune-modulating drugs on severe post-COVID-19 outcomes is mediated by COVID-19 incidence and severity, or whether there is a direct pathway from these drugs to Long COVID or mortality.

## RESULTS

We analyzed electronic health record data from a sample of 3,553 patients with rheumatoid arthritis who were prescribed tocilizumab, sarilumab, anakinra, or baricitinib in 2022. In our sample, 2,622 patients were taking treatment drugs (tocilizumab or sarilumab), while 931 patients were taking control drugs (anakinra or baricitinib). The average age of treatment patients was 57 years, while the average age of comparator patients was 56 years. Most patients were White non-Hispanic (69% in the treatment group, 64% in the comparator group). Treatment patients had an average Charlson Comorbidity Index of 2.5, compared to 3.1 for comparator patients.

We found that prescription to IL-6 modulating drugs, compared to prescription to other biologic agents, was associated with a lower 12-month cumulative risk of mortality (adjusted relative risk (aRR) 0.40, 95% CI 0.27, 0.59), diagnosed Long COVID (aRR 0.42, 95% CI 0.23, 0.78), and probable Long COVID (aRR 0.71, 95% CI 0.61, 0.83). We found that prescription to IL-6 modulating drugs was associated with a lower 12-month cumulative incidence of COVID-19 (aRR 0.71, 95% CI 0.58, 0.86) or severe COVID-19 (aRR 0.42, 95% CI 0.24, 0.72), compared with prescription of other biologic agents.

In our secondary analysis, restricting our sample to only include patients who were prescribed a study drug after acute COVID-19, we did not find a significant controlled direct effect of IL-6 modulating drugs, compared with other biologic agents, on the cumulative risk of mortality (controlled direct effect adjusted relative risk (aRR_CDE_) 0.95, 95% CI 0.39, 2.39), diagnosed Long COVID (aRR_CDE_ 0.53, 95% CI 0.20, 1.41), or probable Long COVID (aRR_CDE_ 0.90, 95% CI 0.75, 1.06), among COVID-19-positive patients (Supplemental Material 1).

## DISCUSSION

We found evidence that prescription to IL-6 modulating drugs (sarilumab or tocilizumab), compared to other biologic agents, was associated with a lower risk of multiple severe post-COVID-19 outcomes (death, Long COVID diagnosis, and probable Long COVID) in a cohort of patients with rheumatoid arthritis. This provides support for the benefit of IL-6 modulating drugs for rheumatoid arthritis patients during the COVID-19 pandemic, particularly patients who may be vulnerable to severe COVID-19. Furthermore, these findings support the role of IL-6 in influencing COVID-19 severity and the development of severe long-term sequelae of COVID-19, such as death or Long COVID.

IL-6 signaling plays a crucial role in mediating systemic inflammation and endothelial function, which are key mechanisms of acute COVID-19 and post-acute sequelae of COVID-19.^47–49^ IL-6 receptor antagonists may interrupt this pathway during acute infection and prevent the long-term consequences of severe infection. In addition, previous studies have consistently found elevated IL-6 levels among Long COVID patients and have hypothesized that IL-6 may mediate long-term neurological symptoms of COVID-19 infection by disrupting T helper 17 and regulatory T cell responses, contributing to sustained inflammatory dysfunction and ultimately leading to fatigue.^10^

We did not find a significant, controlled direct effect of IL-6 modulating drugs, compared with other biologic agents, on post-COVID-19 outcomes among COVID-19-positive individuals, indicating that the protective effects of IL-6 modulating drugs are largely mediated by reduced risk of COVID-19 and reduced COVID-19 severity. In our secondary analysis, we evaluated a subset of our total sample in which the drug start date (new IL-6 medications or comparator) was after acute COVID-19 incidence. While we found a significant protective effect of IL-6 medications on severe post-COVID-19 outcomes in patients who were prescribed IL-6 medications before COVID-19, we found no protective effect in patients prescribed IL-6 medications after COVID-19. Therefore, our findings support that only IL-6 modulating drugs delivered before acute COVID-19, not during or after acute COVID-19, may protect against long-term sequelae of COVID-19.

### Strengths, limitations, and areas for future study

The EHR data source was a considerable strength of this study, as it contained a large sample size of patients and a wide range of covariate information for each patient.^50^ The analysis approach, leveraging Super Learner and targeted maximum likelihood estimation, was a second strength of this study, as it enabled semiparametric evaluation of this research question using a doubly-robust estimation method, minimizing bias and confounding.^19–22,51^

Our findings regarding the protective relationship between IL-6 modulating drugs and acute COVID-19 severity are consistent with previous studies. N3C’s sampling methodology includes a test-negative design of COVID-19 cases and sociodemographically matched controls who seek COVID-19 testing at a testing facility.^16,17^ Our finding that IL-6 modulating drugs were associated with reduced COVID-19 incidence may be a consequence of this sampling methodology and reduced COVID-19 severity in IL-6 patients, as patients who are asymptomatic or have mild COVID-19 may not seek SARS-CoV-2 testing at a healthcare facility. Therefore, we do not assume a causal relationship between IL-6 RA medications and COVID-19 incidence.

The generalizability of N3C is a limitation, as it oversamples patients with high healthcare-seeking behavior, leading to an overrepresentation of patients with multiple comorbidities, who are white, and who are older.^38,40,41^ While our study specifically seeks to evaluate the relationship between IL-6 and Long COVID, the interdependence of inflammation and cytokines complicates these pathways.^3,7,47^ Similarly, the use of biologic agents has myriad effects across immune pathways. There is also a possibility of residual confounding by indication, as patients with rheumatoid arthritis who were prescribed tocilizumab, sarilumab, anakinra, or baricitinib may reflect unique populations. While we sought to account for this baseline imbalance through covariate adjustment and doubly robust estimation, the possibility of residual confounding remains a concern. The low sensitivity of Long COVID diagnosis is a limitation, as Long COVID is rarely diagnosed and documented in EHR due to the wide range of phenotypic manifestations and few treatment options for Long COVID patients.^38,40,52^ We sought to overcome this potential limitation by including probable Long COVID as an additional outcome, which has greater sensitivity than a Long COVID diagnosis but may have less clinical utility. Finally, biomarker data are limited in N3C, which precludes a direct measurement of IL-6 biomarkers. Future studies should include direct biomarker evaluation to determine the mechanistic role of IL-6 in the development of severe post-COVID-19 outcomes.

## CONCLUSIONS

Among patients with rheumatoid arthritis, we found that prescription of IL-6 modulating medications was associated with reduced risk of long-term sequelae of COVID-19, which may be mediated through protection against COVID-19 incidence and severity. This supports the utility of IL-6 modulating drugs for rheumatoid arthritis patients who are at risk of severe COVID-19 and provides indirect support for IL-6 as a mechanistic biomarker of long-term sequelae of COVID-19.

## Supplementary Material

Supplement 1

## Figures and Tables

**Figure 1.**
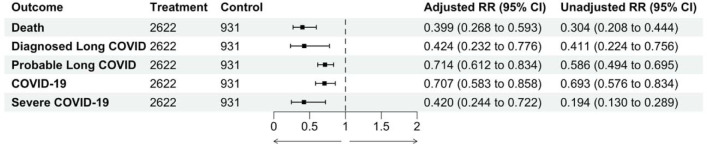
Relationship between IL-6 modulating drugs (tocilizumab or sarilumab) vs. other biologic agents (anakinra or baricitinib) and 12-month cumulative incidence of post-COVID-19 and acute COVID-19 outcomes, among patients with moderate to severe rheumatoid arthritis prescribed a study drug in 2022.

**Table 1.** Patients with moderate to severe rheumatoid arthritis who were prescribed tocilizumab, sarilumab (treatment), anakinra, or baricitinib (control) in 2022 in the National Clinical Cohort Collaborative.

Characteristic	Value	Tocilizumab and Sarilumab	Baricitinib and Anakinra
**Total**		2622 (0.738)	931 (0.262)
**Socioeconomic Variables**	Percent Households Below Poverty Line: mean (SD)	15.37 (5.29)	15.86 (5.45)
	Social Vulnerability Index: mean (SD)	7.34 (1.68)	7.4 (1.75)
**Medical Conditions**	Tobacco Smoker: Count (proportion)	374 (0.143)	120 (0.129)
	Acute Kidney Injury: Count (proportion)	195 (0.074)	197 (0.212)
	Myocardial Infarction: Count (proportion)	128 (0.049)	77 (0.083)
	Congestive Heart Failure: Count (proportion)	233 (0.089)	156 (0.168)
	Heart Failure: Count (proportion)	259 (0.099)	164 (0.176)
	Complicated Diabetes: Count (proportion)	290 (0.111)	166 (0.178)
	Uncomplicated Diabetes: Count (proportion)	436 (0.166)	236 (0.253)
	Diabetes (any): Count (proportion)	505 (0.193)	283 (0.304)
	Chronic Lung Disease: Count (proportion)	782 (0.298)	291 (0.313)
	Other Immunocompromised: Count (proportion)	2109 (0.804)	584 (0.627)
	Charlson Comorbidity Index: mean (SD)	2.46 (2.26)	3.14 (3.07)
	Systemic Corticosteroids: Count (proportion)	1973 (0.752)	693 (0.744)
	Depression: Count (proportion)	528 (0.201)	212 (0.228)
	Bipolar Disorder: Count (proportion)	47 (0.018)	23 (0.025)
	Hypertension: Count (proportion)	1086 (0.414)	475 (0.51)
	Asthma: Count (proportion)	333 (0.127)	123 (0.132)
**Demographics**	Age [years]: mean (SD)	57.01 (19.38)	55.82 (20.65)
	BMI: mean (SD)	32.94 (10.15)	34.37 (10.98)
	Sex: Female	1961 (0.748)	563 (0.605)
**Race/Ethnicity**	Asian Non-Hispanic: Count (proportion)	56 (0.021)	30 (0.032)
	White Non-Hispanic: Count (proportion)	1816 (0.693)	594 (0.638)
	Black or African American Non-Hispanic: Count (proportion)	302 (0.115)	181 (0.194)
	Hispanic or Latino Any Race: Count (proportion)	285 (0.109)	59 (0.063)
**COVID-19**	Vaccination 0–6 months before enrollment: Count (proportion)	153 (0.058)	48 (0.052)
	Vaccination 6–12 months before enrollment: Count (proportion)	341 (0.13)	114 (0.122)
**Healthcare Utilization**	Pre-baseline Utilization Rate: mean (SD)	2.9 (3.18)	2.73 (3.38)
	At least 1 interaction after inclusion: Count (proportion)	2469 (0.942)	851 (0.914)

## Data Availability

All analytic code and data are available in the N3C Enclave by request. Access to the N3C Data Enclave is managed by NCATS (https://ncats.nih.gov/research/research-activities/n3c/resources/data-access). Interested researchers must first complete a data use agreement, and next a data use request, in order to access the N3C Data Enclave. Once access is granted, the N3C data use committee must review and approve all use of data and the publication committee must approve all publications involving N3C data.
