# Supplementary material for: IL-6 Receptor Antagonists and Severe Post-COVID-19 Outcomes: An Emulated Target Trial
Source: medRxiv. 2026 Mar 2:2026.02.27.26347274. Preprint. [Version 1] doi: 10.64898/2026.02.27.26347274 (PMC12976917; doi:10.64898/2026.02.27.26347274)

**SUPPLEMENTAL MATERIALS**

Supplemental Material 1. Relationships between IL-6 modulating drugs (tocilizumab or sarilumab) vs. other biologic agents (anakinra or baricitinib) and the 12-month cumulative incidence of post-COVID-19 outcomes among type 2 diabetes mellitus patients prescribed a study drug in 2022 with documented acute COVID-19 before the drug start date.
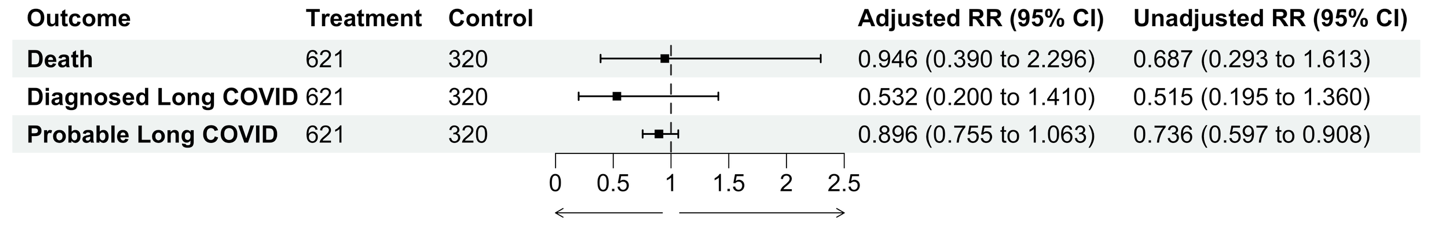

Supplement: Supplement 1 [file media-1.docx]
